# Completeness of tuberculosis (TB) notification: inventory studies and capture-recapture analyses, six European Union countries, 2014 to 2016

**DOI:** 10.2807/1560-7917.ES.2020.25.12.1900568

**Published:** 2020-03-26

**Authors:** Masja Straetemans, Mirjam I Bakker, Sandra Alba, Christina Mergenthaler, Ente Rood, Peter H Andersen, Henrieke Schimmel, Aleksandar Simunovic, Petra Svetina, Carlos Carvalho, Outi Lyytikäinen, Ibrahim Abubakar, Ross J Harris, Csaba Ködmön, Marieke J van der Werf, Rob van Hest

**Affiliations:** 1 KIT Royal Tropical Institute, Health Unit, Amsterdam, the Netherlands; 2 Statens Serum Institute, (National Institute for Public Health), Department of Infectious Disease Epidemiology and Prevention, Copenhagen, Denmark; 3 National Institute for Public Health and the Environment (RIVM), Centre for Infectious Disease Control, Bilthoven, the Netherlands; 4 Croatian Institute of Public Health, Infectious Disease Epidemiology Service, Zagreb, Croatia; 5 University Clinic of Pulmonary Diseases and Allergy Golnik, Department of Tuberculosis, Golnik, Slovenia; 6 University of Porto, Institute of Biomedical Sciences Abel Salazar (ICBAS), Multidisciplinary Unit for Biomedical Research (UMIB), Porto, Portugal; 7 Portuguese Northern Regional Health Administration, Public Health Department, Porto, Portugal; 8 National Institute for Health and Welfare (THL), Helsinki, Finland; 9 Institute for Global Health, University College of London (UCL), London, United Kingdom; 10 Public Health England (PHE), Statistics Unit, London, United Kingdom; 11 European Centre for Disease Prevention and Control (ECDC), Stockholm, Sweden; 12 Department of Tuberculosis Control, Regional Public Health Service Groningen and Fryslân (GGD), Groningen, the Netherlands; 13 Department of Pulmonology and Tuberculosis, University Medical Centre Groningen (UMCG), Groningen, the Netherlands

**Keywords:** tuberculosis, surveillance, epidemiology, statistics, Croatia, Denmark, Netherlands, Portugal, Slovenia, Finland

## Abstract

**Background:**

Progress towards the World Health Organization’s End TB Strategy is monitored by assessing tuberculosis (TB) incidence, often derived from TB notification, assuming complete case detection and reporting. This assumption is unlikely to hold in many settings, including European Union (EU) countries.

**Aim:**

We aimed to assess observed and estimated completeness of TB notification through inventory studies and capture–recapture (CRC) methodology in six EU countries: Croatia, Denmark, Finland, the Netherlands, Portugal Slovenia.

**Methods:**

We performed record linkage, case ascertainment and CRC analyses of data collected retrospectively from at least three national TB-related registers in each country between 2014 and 2016.

**Results:**

Observed completeness of TB notification by inventory studies was 73.9% in Croatia, 98.7% in Denmark, 83.6% in Finland, 81.6% in the Netherlands, 85.8% in Portugal and 100% in Slovenia. Subsequent CRC analysis estimated completeness of TB notification to be 98.4% in Denmark, 76.5% in Finland and 77.0% in Portugal. In Croatia, CRC analyses produced implausible results while in the Netherlands and Slovenia, it was methodologically considered not meaningful.

**Conclusion:**

Inventory studies and CRC methodology suggest a TB notification completeness between 73.9% and 100% in the six EU countries. Mandatory reporting by clinicians and laboratories, and cross-checking of registers, strongly contributes to accurate notification rates, but hospital episode registers likely contain a considerable proportion of false-positive TB records and are thus less useful. Further strengthening routine surveillance to count TB cases, i.e. incidence, accurately by employing record-linkage of high-quality TB registers should make CRC studies obsolete in EU countries.

## Introduction

In 2014, the World Health Organization (WHO) published the Action Framework towards TB elimination in low-incidence countries, and in 2016, the WHO Regional Office for Europe (WHO/Europe) published the Roadmap to implement the tuberculosis action plan for the WHO European Region 2016-2020: Towards ending TB and multidrug-resistant tuberculosis. They outline blueprints to carry out the WHO’s global End TB Strategy in Europe and to reach the sustainable development goal (SDG) target for tuberculosis (TB) [1-3]. Key strategic targets include reduction of global TB incidence by 80% in 2030 and 90% in 2035, compared with 2015 [4,5]. In the WHO European Region, the targets include a 25% reduction in TB incidence rate by the year 2020 compared with 2016 [3]. TB incidence can be derived from TB notification rates, assuming complete case detection and reporting. This is considered a strong assumption unlikely to hold in many settings, including European Union (EU) countries. Several studies across the EU/EEA, e.g. those from France, Spain, Italy and Romania, have revealed high rates of under-reporting [6,7].

Inventory studies (IS) are a widely accepted methodology to study the level of under-reporting of TB cases [8]. To determine completeness of TB notification, TB IS compare the number of cases meeting standard case definitions and recorded in multiple TB-related registers, such as laboratory registers or hospital episode registers, with the cases notified to local and national authorities [9,10]. For this comparison, record-linkage methodologies are used. Subsequently, through capture–recapture (CRC) analysis, the number of cases unknown to all registers can be estimated, thereby providing an estimate of the completeness of TB notification [11].

At the time of the study and writing this article, the United Kingdom (UK) was still part of the EU and only two EU countries, the Netherlands and the UK, had assessed the level of TB under-reporting using IS/CRC studies on a national basis [11,12]. In 2016, the European Centre for Disease Prevention and Control (ECDC) commissioned an IS/CRC project to estimate TB under-notification in six to nine EU countries and expand the evidence base of the methodology.

The objective of this study was to assess observed and estimated TB notification completeness in EU countries, selected after an eligibility and feasibility appraisal, and determine whether data collected at national level reflect the real TB situation in these countries.

## Methods

### Study design

This study used inventory studies and capture-recapture analyses as per the methodology outlined by Van Hest and the WHO [6,8].

### Country selection process

To select the six most appropriate and representative EU and European Economic Area (EEA) countries for the study, we followed a predefined selection procedure that was developed by the consortium commissioned to perform this work and agreed upon with ECDC during the project kick-off meeting in June 2016. First, eligibility and feasibility for IS and more specifically CRC studies was assessed by an 11-item tick-box questionnaire that was distributed to the TB operational contact points for epidemiology at ECDC and WHO/Europe TB surveillance network meeting in Bratislava in June 2016 (Table 1). Non-responders and countries not present at this meeting were invited by email to return the questionnaire by 1 August 2016. Countries who had not responded by 29 July received a reminder email from ECDC to fill out the form, but this did not yield any additional responses. Twenty forms were received from 31 countries (28 EU and 3 EEA): Belgium, Bulgaria, Croatia, Czech Republic, Denmark, Estonia, Finland, France, Germany, Ireland, Malta, the Netherlands, Poland, Portugal, Romania, Slovakia, Slovenia, Spain, the UK and Norway. Four countries, France, Malta, the UK and Norway, indicated they did not want to participate. Germany and Ireland indicated they did not have a third case-based register or national data which are essential requirements.

**Table 1:** Tuberculosis inventory study eligibility and feasibility appraisal questionnaire with simple scoring system, European Union and European Economic Area countries, 2016

Question number	Question	Score^a^
1	Does your country have an electronic case-based TB notification database?	Essential
2	Are these the data you report to ECDC/TESSy?	1
3	Is TB notification mandatory in your country?	2
4	Only mandatory for the doctor or also for other, e.g. laboratory?	2
5	Does your country have other electronic case-based TB registers available?	Essential
6	Are standard TB case definitions used in these registers?	2
7	Do these registers contain variables that can be used for record linkage?	Essential
8	Does the NTP already routinely link certain TB registers?	1
9	Does the NTP have professionals that can assist in this study, e.g. data managers, statisticians?	2
10	Does your country have legal issues hindering capture–recapture studies?	Essential
11	Would your country be interested in participating?	Essential

ECDC: European Centre for Disease Prevention and Control; NTP: National TB Programme; TB: tuberculosis; TESSy: The European Surveillance System.

^a^ Scoring based on conditions considered essential, important (2 points), or nice (1 point) to have or not to have.

To obtain detailed information regarding TB-related registers, data availability, data access and data quality, the 14 countries identified in this rapid eligibility and feasibility assessment were contacted by 1 hr telephone or Skype calls, with a follow-up call or email for some countries. Subsequently, a simple scoring system was applied to ensure a consistent and systematic selection of countries (Table 1). A matrix of geographical location, economic status, TB incidence and proportion of foreign-born TB patients was also prepared to ensure diverse representation of countries. After the eligibility and feasibility appraisal, six countries, Croatia, Denmark, Finland, the Netherlands, Portugal and Slovenia, were selected for the IS and CRC analyses, and country-specific protocols were developed.

### Case definition and inclusion criteria

This study included all cases recorded as active pulmonary or extrapulmonary TB, new as well as previously treated or diagnosed cases, in the various registers of the six EU countries in the selected study year. Double entries in each of the registers, cases caused by non-TB mycobacteria (NTM) or cases later diagnosed as not having TB were excluded. TB diagnosis was either: (i) bacteriologically confirmed by a positive culture for *Mycobacterium tuberculosis* complex (gold standard), (ii) bacteriologically confirmed by a positive auramine or Ziehl-Neelsen stained respiratory or non-respiratory sample and/or a positive PCR for *M. tuberculosis* complex, or (iii) based on clinical, radiological, histopathological or epidemiological findings with an intention to treat. Hospital episode records were included when coded with International Code of Disease (ICD)-9 code 010–018 or ICD-10 code A15-A19 [13,14].

### Inclusion and period of data collection

TB cases with a date of notification, mycobacteriological confirmation, first day of hospital admission or death between 1 January and 31 December of the same study year were included. Study years varied in the countries between 2014 and 2016 (Table 2). To allow for correction of late positive results from laboratory diagnostics or late notification, the period of data collection was from 6 months before the year of study until 3 months after (6 months after in Portugal).

**Table 2:** Demographics and main findings from inventory studies and capture–recapture analyses, six selected European Union countries, 2014–2016

Country (study year)	Croatia (2015)	Denmark (2015)	Finland (2014)	Portugal (2015)	The Netherlands (2014)	Slovenia (2016)
**Number of inhabitants^a, b^**	4,225,316	5,659,715	5,451,270	10,374,822	16,829,289	2,064,188
**Region of Europe^c^**	Southern	Northern	Northern	Southern	Western	Southern
**Gross domestic product per capita^a, b^**	10,600 EUR	48,000 EUR	37,600 EUR	17,400 EUR	39,800 EUR	19,500 EUR
**TB notifications TESSy^a^/100,000 (n^d^)**	11.6 (486)	6.3 (357)	4.8 (263)	21.0 (2,178)	4.8 (814)	5.7 (118)
**TB notifications national TB register**	489	379	260	2,182	814	118
**Estimated TB incidence^a^/100,000 (n^d^)**	13.0 (560)	6.5 (370)	5.3 (290)	23.0 (2,400)	5.8 (980)	6.5 (140)
**Foreign-born (%)/unknown (%)^a^**	15.2/32.9	67.8/0.0	33.1/1.5	16.7/0.1	73.8/0.0	36.4/0.0
**Geographic coverage**	National	National	National	Islands excluded	National	National
**National registers used**	1. TB Notification Register (NTR) 2. Mycobacterial Reference Laboratory database (NRL) 3. Hospital discharge register	1. TB notification register (MIS2)^e^ 2. Mycobacterial reference laboratory and Danish microbiology database (MiBa) 3. Hospital discharge register (National Patient Register)	1. National Infectious Disease Register (NIDR/TTR) 2. Hospital discharge register (Hilmo) 3. Primary health center discharge register (AvoHilmo) 4. Death register	1. TB register (Sistema de Vigilância da Tuberculose - SVIG TB) 2. Notifiable disease surveillance system (Sistema Nacional de Vigilância Epidemiológica - SINAVE) 3. Hospital discharge register (Grupos de Diagnóstico Homogéneos - GDH)	1. The Netherlands Tuberculosis Register (NTR) 2. Mycobacteriology reference laboratory register 3. Hospital discharge register	1. TB notification register 2. Laboratory register 3. Hospital register 4. Mortality register
**Record linkage**	Probabilistic: first name, family name (Jaro-Wrinkler distance algorithm)	Deterministic: national identity code	Deterministic: national identity code	Probabilistic: date of birth, sex, place of residence	Deterministic: (relaxed) combination of major and minor proxy identifiers^f^	Deterministic: full name of patient
**Statistical models**	Poisson log-linear regression model 3-source data	Poisson log-linear regression model 3-source data	Poisson log-linear regression model 3-source data and 4-source data	Poisson log-linear regression model 3-source data	CRC not feasible due to complete overlap of two registers and one considered poor data quality	CRC not feasible because of almost complete overlap
**Results of IS: observed completeness of notification**	73.9% (489/662)	98.7% (379/384)	83.6% (260/311)^g^	85.8% (1,997/2,328)	81.6% (814/998)	100% (118/118)
**Results of CRC: number of estimated unobserved cases (95% CI)**	Considered implausible result	*1* (95% CI: 0–3)	117 (95% CI: 26–515)^h^/20 (95% CI: 2–234)^i^	266 (95% CI 198–358)^j^	Not assessed	Not assessed
**Estimated completeness of notification in %**	Not assessed	98.4% (95% CI 97.9–98.7)	76.5% (95% CI 63.7–81.3)^k^	77.0% (95% CI 74.3% to 79.1%)^j^	Not assessed	Not assessed
**Main challenge identified**	81% (140/173) unnotified TB cases were only available in hospital register Possibly presence of false-positive TB cases in hospital discharge register Possible unmeasurable strong 3-way dependency	Direct referral of cases from one source to another results in pairwise dependence that can be handled by log-linear models.	Many of the 313 (573–260) Hilmo and Avohilmo cases not notified are likely to be false positive	Probably false-positive TB cases in public health register and hospital discharge register Admission to hospital is likely related to severity of disease, potentially violating the assumption of homogeneous capture probabilities.	A check with non-TB mycobacteria register and latent TB infection data filtered 14 hospital-only cases out; the remaining 184 cases were registered in hospital discharge only which is expected to contain many false-positive records.	A priori notification and laboratory records expected to be highly interdependent, but other registers also appeared to be interdependent because of: (i) Majority of TB patients starting treatment in hospital; (ii) Regular mortality audits with hospital register’s cause of death data
**Conclusion**	Impossible to interpret CRC analyses without following up on clinically diagnosed TB patients only available in hospital register.	Systematic validation of notification and laboratory register led to improved accuracy of data.	Primary health centre discharge data were found to be unreliable and follow-up of false positives is needed.	Number of unobserved cases is likely to be higher than previously thought but, due to likely presence of false-positive TB cases in hospital discharge registers, lower than estimated in this study.	Proportion of under-notification in the Netherlands in 2014 pending availability of time and resources necessary to investigate non-matching hospital-only TB cases.	Completeness of TB notification is high; only small percentage is culture negative.

CI: confidence interval; CRC: capture-recapture; IS: inventory studies; TB: tuberculosis; n: number.

^a^ For country-specific study year.

^b^ Source: Eurostat.

^c^ Based on United Nations geoscheme [33].

^d^ Source: Tuberculosis surveillance and monitoring in Europe 2018; absolute number as reported to TESSy.

^e^ MIS2 Meldesystemet for Infektions Sygdomme version 2.

^f^ Major proxy identifiers include: year or full date of birth; sex, four-digit postal code, and minor proxy identifiers include date of notification, date of first bacteriology culture sample and date of hospital admission.

^g^ After adjustment for 90% suspected false-positive TB cases.

^h^ Excluding death register and assuming that 10% of cases in primary health centre and hospital discharge register are true TB cases.

^i^ Including death register, excluding primary health centre discharge register, assumption 10% of Hilmo cases are true TB cases.

^j^ After probabilistic matching and excluding possible cases.

^k^ Four-source model under assumption that 10% of Hilmo and Avohilmo-only cases are true TB cases.

### Sources used to identify tuberculosis cases

The national electronic databases with case-based records of registered TB cases used in the IS in the six EU countries are listed in Table 2.

### Record linkage

Two different approaches were used to link records of the same individual in different databases [8]. Deterministic record linkage, identical identifiers across datasets for a match, was performed when a unique identifier was available (Denmark, Finland, Portugal and Slovenia). Relaxed deterministic record linkage was used in the Netherlands, allowing for slight differences between the combination of identifiers in the various registers. Probabilistic record linkage, based on a likelihood score of a match [8], was used when a combination of proxy-identifiers, e.g. initials of the name, full date of birth, full postcode and sex of the patient, was available (Croatia, Portugal) (Table 2). In Portugal probabilistic record linkage was performed after deterministic record linkage provided unrealistic results due to errors in the data sources.

### Analysis

Observed TB notification completeness was calculated as the proportion of TB cases notified among the total number of TB cases observed after record linkage through the retrospective ISs. Estimated TB notification completeness were obtained through CRC analysis. The preferred CRC method entails log-linear modelling of at least three possibly incomplete, partially overlapping and preferably, but not necessarily, independent registers. Three-source log-linear modelling is less compromised by violation of the underlying modelling assumptions (e.g. a closed and homogeneous population; independence of registers; absence of false-positive cases) [6,8,15-19]. CRC aims to estimate the unobserved number of TB cases from the information provided by the overlap between the linked registers. The unobserved number of TB cases were modelled based on rates of presence in each register (main effects) and pairwise dependencies (interactions) between registers; the latter were estimated as incidence rate ratios (IRRs), where estimates > 1 indicated a positive dependence between registers. Three linked registers provide eight different possible models ranging from containing no dependencies (the base model), any possible combination of the three pairwise dependencies, to the saturated model, containing all dependencies. The interaction between all three registers cannot be estimated and must be assumed absent. Selection of the preferred model (and thus the estimate of unobserved TB cases) was determined by balancing model fit with parsimony, based on Akaike Information Criterion (AIC) scores, coupled with knowledge of epidemiological plausibility, i.e. consistency with prior reports or estimates [12]. The estimated completeness of notification was the proportion of TB cases notified of the estimated total number of TB cases [6,8,15-19]. Poisson log-linear modelling of three independent registers was done in Croatia, Denmark and Portugal. For Finland, modelling was extended to four sources, incorporating proportions of false-positive TB cases, based on external evidence. CRC was considered not feasible in the Netherlands and Slovenia because of complete overlap of respectively two and three registers.

### Study permission

National medical-ethical authority permission was obtained (Finland (Tietosuojavaltuutetun toimisto: THL/112/6.02.00/2017), the Netherlands (Data Protection Committee of the Netherlands Tuberculosis Register Number: 04-2017) and Portugal (Comissão Nacional de Protecção de Dados (CNPD) Processo no: 11543/2017)) or not required (Denmark, Croatia and Slovenia).

## Results

In Croatia, record linkage of three national registers (notifications (489 cases), reference laboratory (334 cases), and hospital episodes (476 cases)) resulted in a total of 662 registered TB cases, translating into an observed completeness of notification of 73.9% (489/662). Of the 173 TB cases not notified, 140 (81%) were only known to the hospital episode register (Figure). In CRC analysis, AIC scores favoured the saturated model, which estimated an implausibly high number of unobserved TB cases (1,705; 95% confidence interval (CI): 707–4,114).

**Figure:**
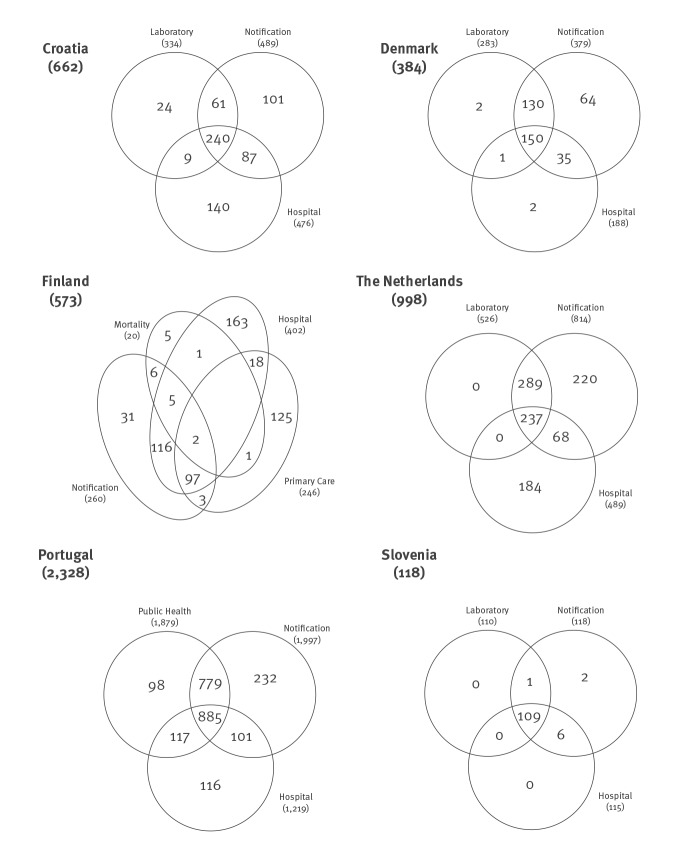
Schematic view of the registered number of tuberculosis (TB) cases after record linkage of three TB-related registers, six selected European Union countries, 2014–2016

The circles show the number of patients in each registration and their overlap (for clarity, the Venn diagram is not projected to scale of the number of tuberculosis (TB) cases in each cell).

Finland Venn diagram shows the number of TB cases based on the preferred four-source record-linkage adjusted for suspected 90% false positive TB cases. Portugal Venn diagram shows the number of TB cases based on probabilistic linkage and excluding possible TB cases.

In Denmark, record linkage of three national registers (notifications (379 cases), reference laboratory (283 cases), and hospital episodes (188 cases)) resulted in a total of 384 registered TB cases, translating into an observed completeness of notification of 98.7% (379/384). Of the five TB cases not notified, two were only known to the hospital episode register, two to the laboratory register only and one to both registers. CRC analysis selected a model with one interaction (between the laboratory and hospital episode registers), estimating one unobserved TB case, and resulting in an estimated completeness of notification of 98.4% (379/385) (95% CI: 97.9–98.7).

In Finland, complete overlap between the reference laboratory and the notification registers was expected due to automatic registration of the laboratory results. Record linkage of the notification register (260 cases) with the hospital episode (402 cases) and primary care episode (246 cases) registers resulted in a total of 569 registered TB cases, translating into an observed completeness of notification of 45.7% (260/569). After record linkage with a fourth register, the mortality register (20 cases), 573 TB cases were observed, translating into an observed completeness of notification of 45.4% (260/573). A large part of the TB cases not notified (573–260 = 313) appeared either only in the hospital register (52% (163/313) or in the primary care register (40% (125/313). Unpublished findings from a Finnish pilot study, brought to our attention during implementation of our research, in which hospital records of TB patients with ICD-10 codes A15-A19 were reviewed in detail, found that only 10% were true, notifiable, TB cases (personal communication, Hanna Soini, August 2019). Since it was not in the scope of our study to review clinical records, we assumed 90% of the hospital or primary care only cases to be false-positive. After adjusting for these suspected false-positive TB cases, the observed completeness of notification was 83.6% (260/311). CRC analysis on the unadjusted data preferred the saturated model, which estimated an implausibly high number of unobserved TB cases in Finland in 2014, more than 15 times the number of observed TB cases. Several other models were considered, as described in detail elsewhere (country report for Finland available upon request from ECDC). The four-source CRC was the preferred analysis because it provided a very similar estimate to the three-source CRC which included the mortality register, and it corroborated the assumption that many of the Hilmo-only or Avohilmo-only cases are false positive TB cases. The preferred four-source CRC analysis on the data adjusted for the 90% suspected false-positive TB cases estimated 29 unknown TB cases, translating into an estimated completeness of notification of 76.5% (95% CI: 63.7–81.3%) (260 notified cases/340 estimated true TB cases).

In the Netherlands, record linkage of three national registers (notifications (814 cases), reference laboratory (526 cases), and hospital episodes (489 cases)) resulted in a total of 998 registered TB cases in 2014, translating into an observed completeness of notification of 81.6% (814/998). All culture-confirmed laboratory TB cases were notified (observed completeness of notification of 100%). Because of this complete overlap two linked registers remained, with all TB cases not notified (n = 184; after excluding NTM (10 cases) and latent TB infections (4 cases)) only known to the hospital episode register, indicating that 49.0% (489/998) of the TB cases in the Netherlands in 2014 were (initially) hospitalised. Scrutinising the individual patient records of the cases only available in the hospital episode registry to assess whether they had signs, symptoms or diagnostic test results suggestive for TB, or whether treatment was initiated and completed, was not possible for confidentiality, logistical and financial reasons. A CRC analysis was methodologically not considered meaningful due to the complete overlap of the notification this would be a two-source CRC (see Discussion) [20]

In Portugal, three TB registers were identified in 2015: the national TB programme register (2,086 cases), the public health register (1,874 cases) and the hospital episode register (1,358 cases). Case ascertainment was 3,561 (deterministic linkage; all TB cases); 2,914 (deterministic linkage; excluding ‘possible’ TB cases); 2,786 (probabilistic linkage; all TB cases); and 2,328 TB cases (probabilistic linkage; excluding ‘possible’ TB cases); with an observed completeness of notification of 60.2% (2,144/3,561), 64.6% (1,883/2,914), 80.0% (2,228/2,786), and 85.8% (1,997/2,328), respectively. For all datasets, a parsimonious CRC model with two (positive) pairwise interactions (national TB register*public health register and public health register*hospital episode register) was selected. The preferred model estimated completeness of notification at 77.0% (95% CI: 74.3%–79.1%), after probabilistic record linkage and excluding the ‘possible’ TB cases (1,997/(2,328 observed TB cases and 266 unobserved TB cases)).

In Slovenia, a strong positive interaction between the notification (118 cases), reference laboratory (111 cases) and hospital episode (115 TB cases) registers was expected, as all these services are mainly concentrated in one university hospital. Record linkage of these registers with the mortality (19 cases) register resulted in a case ascertainment of 119 TB cases. Excluding one person with a positive culture of *M. tuberculosis* starting treatment abroad and assumed notified there, 118 TB cases were registered in Slovenia in 2016, translating into a completeness of notification of 100%. Due to the almost complete overlap of the registers used, CRC analysis was methodologically considered not meaningful.

## Discussion

Observed completeness of TB notification was calculated to be 73.9% in Croatia, 98.7% in Denmark, 83.6% in Finland, 81.6% in the Netherlands, 85.8% in Portugal, and 100% in Slovenia. Subsequent CRC analyses estimated completeness of TB notification to be 98.4% in Denmark, 76.5% in Finland and 77.0% in Portugal. In the other three countries, CRC analysis gave implausibly high estimates of unobserved TB cases (Croatia) or was not performed because it was methodologically not considered meaningful (the Netherlands, Slovenia). In the Netherlands and Slovenia only two databases could be used and using two-source capture–recapture models for epidemiological data often violates the underlying capture–recapture assumptions, resulting in biased estimates and is thus not preferred [20]. According to the latest ECDC/WHO Europe TB surveillance and monitoring report, completeness of notification was estimated as 88.5%, 94.3%, 90.8%, 88.9%, 87.4%, and 84.3% for Croatia, Denmark, Finland, the Netherlands, Portugal and Slovenia, respectively [21].

In monitoring progress in TB control, the ultimate aim is to count TB cases (incidence) accurately through routine surveillance. When routine surveillance is not robust, alternative methods can be used to assess completeness of notification, as described elsewhere [22]. One such method is a prevalence survey (active case finding) [23]. However, prevalence surveys are costly and laborious and can only be justified in countries where many cases are thought to be missed and the expected smear positive TB prevalence is high enough (≥ 100 per 100,000 population) to obtain an accurate estimate with a reasonable sample size [23,24].

Surveillance by notification may result in inaccurate incidence rates because of under-ascertainment of the true number of cases or over-ascertainment due to false-positive cases [24]. Standardised means are needed to evaluate and adjust incidence rates [22]. IS and CRC analysis are such methods but can be difficult to implement, even in resource-rich countries [6]. We encountered various challenges when assessing completeness of TB notification in the six EU countries through retrospective IS and CRC methodology: (i) high interdependency between notification and laboratory registers (as a result of improved surveillance, sometimes by mandatory reporting, automatic record linkage, and routine cross-checking of registers); (ii) false-positive TB cases in hospital episode (and related) registers; (iii) privacy issues and aggregated data collection, preventing the use of prescriptions and health insurance registers; and (iv) selection of the CRC model. A comprehensive discussion of violation of underlying CRC assumptions can be found elsewhere [6,18,19].

Previous regional and national IS and CRC studies in EU countries often used the notification, reference laboratory and hospital episode registers, assuming absence of major interdependencies [6,11,12,25,26]. In recent years, many EU countries made it mandatory for the laboratory to report positive bacteriological TB test results, in addition to notification by the diagnosing clinician. This can result in a (near) complete overlap between these registers, as observed in Denmark, the Netherlands and Slovenia. Often, notification and reference laboratory registers are maintained within the same institute, with routine cross-checks between registers and sometimes with (in)direct access to other TB-related registers as well. The assumption that notification and reference laboratory registers in EU countries are relatively independent should be judged critically. In some countries, such as Denmark and Slovenia, where, by convention, almost all TB patients are (initially) hospitalised, a (near) complete overlap and interdependence between the hospital and notification registers was observed. Likewise, negative dependencies are expected when the hospital database includes clinically diagnosed cases, which by definition do not appear in the laboratory register, such as in Croatia. Violation of the assumption of homogenous capture probabilities can lead to biased estimates.

The two previous national IS and CRC studies in EU countries suggested a considerable proportion of false-positive TB cases in hospital episode registers, possibly coding a differential diagnosis upon admission or a presumptive diagnosis upon discharge [11,12]. The diagnosis TB, a disease with a sometimes prolonged diagnostic pathway, can be withdrawn later, e.g. due to absence of positive laboratory results for TB, results indicating NTM, or a final diagnosis other than TB. However, hospital episode registers are not dynamic, with continuous updates and corrections of the ICD codes. A retrospective study conducted in Turku and Tampere university hospitals in Finland (2014–2016), scrutinising the local hospital episode registers to assess whether patients were correctly coded with an ICD code for active TB, found 90% of the records to be false-positive (i.e. patients ultimately not diagnosed with and offered complete treatment for TB) (personal communication, Hanna Soini, August 2019). Based on this knowledge, a correction for (assumed) false-positive TB cases could be made, resulting in a dramatically reduced and more realistic CRC estimate of the unregistered TB cases in Finland. In Portugal, similar outcomes were obtained after correction for ‘possible’ TB cases. When (nearly) all records only known to the hospital episode register in the Netherlands were considered false-positive, the observed completeness of notification was (nearly) 100% and, as a result of this correction, 37.5% of the TB cases in the Netherlands were (initially) hospitalised in 2014. This is in line with historical observations that around one third of the TB patients in the Netherlands are hospitalised for more than one week, 247 cases (30%) reported through routine surveillance in 2014 [27]. In Croatia and the Netherlands, comparison of the hospital episode records with clinical information was not possible. Routine review of national hospital episode registers against patient records, in consultation with the clinicians, e.g. half a year after entry, could possibly increase the proportion of true-positive TB cases, possibly reducing over-estimation of the number of TB cases.

Alternative registers that could be used in some EU countries are mortality registers, when not nested in other registers. Other registers include health insurance registers and prescription registers for anti-TB medication, most specifically pyrazinamide, since other first-line drugs are also prescribed for different conditions, such as latent TB infection and treatment of NTM. Possible problems with these registers could be collection of only aggregate data, unsuitable for record linkage, or unavailability of data due to privacy regulations, such as the EU General Data Protection Regulation (GDPR) [28], which came into effect on 25 May 2018. Prescription registers could still be used for triangulation of IS and CRC results through pharmaco-epidemiology by estimating the number of TB cases based on the daily dose equivalent of pyrazinamide [29]. In our study, a limited number of registers were available for linkage: mortality registers only contained few TB-related records and prescription and health insurance registers were not suitable in existing formats or under current regulations.

In some studies, the saturated CRC model, i.e. the model incorporating all pairwise interdependencies but also the highest order interdependency between all registers which cannot be controlled, was preferred based on the lowest AIC [17]. The literature generally recommends extreme caution when this model is chosen as one or more assumptions underlying the CRC analysis are likely violated, making statistical inferences unreliable [18]. Further work would be beneficial, including alternative structural models for the data-generating process that do not rely on log-linear assumptions [30], Bayesian models incorporating prior uncertainty on proportions of false-positive cases [31], data from multiple years to account for individuals observed in different years, or the incorporation of covariate data [32], as attempted in Portugal.

Given the intrinsically complex nature of TB epidemiology, variations in diagnoses (e.g. bacteriologically or clinically confirmed), socioeconomic background and disease severity, the assumption of homogenous probabilities of observations is unlikely to hold. Log-linear models may partially incorporate population heterogeneity, especially if relevant covariate information explaining variation is available, but the problem is likely persistent to some extent and a limitation of CRC [18]. The limited number of TB registers available and the absence of data to assess variations in individual detection probabilities in different countries did not allow to identify how heterogeneity affects population estimates across different settings. Hence, the accuracy of the final population estimates could only be assessed based on expert opinion, critical scrutiny of the nature of various sources and the quality of the data used in the analyses.

Another limitation of this study is that the six countries were selected after an eligibility and feasibility appraisal, with likely selection bias, limiting extrapolation of the results to other EU countries. The six countries selected were predominantly countries with a small population, a limited number of TB cases and a well-organised TB notification and control system.

## Conclusion

Our analysis suggests that only in Denmark and Slovenia the assumption that TB notification reflects TB incidence is likely to be true. In these countries, the estimated number of unobserved TB cases was minimal (Denmark) or expected to be minimal because of complete overlap of TB cases between registers (Slovenia). In the other countries, observations and estimates were more difficult to interpret.

Mandatory reporting by both clinicians and laboratories, and cross-checking of registers, strongly contributes to accurate notification rates but hospital episode registers likely contain a considerable proportion of false-positive TB records and more scrutiny is needed. Further strengthening routine (computerised) infectious disease surveillance systems to count TB cases (incidence) accurately, by structurally employing electronic record linkage of high-quality TB registers, should make CRC studies obsolete in EU countries.
